# Myocardial adaptation as assessed by speckle tracking echocardiography after isolated mitral valve surgery for primary mitral regurgitation

**DOI:** 10.1007/s10554-020-02065-3

**Published:** 2020-10-13

**Authors:** Muhammed Gerçek, Lothar Faber, Volker Rudolph, Henrik Fox, Thomas Puehler, Hazem Omran, Lisa Katharina Wolf, Lech Paluszkiewicz, Andreas M. Zeiher, Kavous Hakim-Meibodi, Jan Gummert, Zisis Dimitriadis

**Affiliations:** 1 Clinic for General and Interventional Cardiology/Angiology, Herz- Und Diabeteszentrum NRW, Ruhr-Universität Bochum, Georgstraße 11, 32545 Bad Oeynhausen, Germany; 2 Clinic for Thoracic and Cardiovascular Surgery, Herz- Und Diabeteszentrum NRW, Ruhr-Universität Bochum, Bad Oeynhausen, Germany; 3 Heart Failure Department, Herz- Und Diabeteszentrum NRW, Ruhr-Universität Bochum, Bad Oeynhausen, Germany; 4 Department of Cardiac and Vascular Surgery, University Medical Center Schleswig Holstein, Campus Kiel, Kiel, Germany; 5 Department of Cardiology, University Hospital Frankfurt, Frankfurt am Main, Germany

**Keywords:** Mitral valve surgery, Strain analysis, Myocardial adaptation

## Abstract

The risk of left ventricular (LV) and right ventricular (RV) maladaptation after surgery for isolated primary mitral regurgitation (PMR) is poorly defined. We aimed to evaluate LV and RV contractile function using speckle-tracking analysis alongside with quantification of exercise tolerance in patients with PMR after mitral valve surgery. All consecutive patients with symptomatic PMR undergoing mitral valve surgery between July 2015 and May 2017 were prospectively enrolled. Sequential echocardiographic studies along with clinical assessment were performed before and three months after surgery. Mean age in 138 patients was 65.8 ± 12.7 years, 48.2% (66) of whom were female. Mean LV ejection fraction decreased from 57 ± 12% to 50 ± 11% (p < 0.001), LV global longitudinal strain deteriorated from −19.2 ± 4.1% to −15.7 ± 3.8% (p < 0.001), and mechanical strain dispersion increased from 88 ± 12 to 117 ± 115 ms (p = 0.004). There was a reduction in tricuspid annulus plane systolic excursion from 22 ± 5 mm to 18 ± 4 mm (p < 0.001), as well as a slight deterioration of RV free wall mean longitudinal strain from −16.9 ± 5.6% to −15.7 ± 4.1% (p = 0.05). The rate of moderate to severe tricuspid regurgitation significantly decreased (p < 0.005). Regarding exercise tolerance, the New York Heart Association class improved (p < 0.001) and the walking distance increased (p < 0.001). During mid-term follow up after surgery for PMR, a deterioration of LV and RV contractile function measures could be observed. However, the clinical status, LV dimensions, and concomitant tricuspid regurgitation improved which in particular imply more effective RV contractile pattern.

## Introduction

Primary mitral regurgitation (PMR) due to mitral valve degeneration is the most common etiology in patients undergoing mitral valve surgery [1]. Surgical mitral valve repair or replacement, if repair is unfeasible, is the treatment of choice in case of symptomatic severe PMR [2].

Yet, patients with mitral regurgitation are often referred too late for surgery due to alleged preserved left ventricular (LV) function in echocardiographic controls [3]. Due to the load dependence of standard echocardiographic parameters which are used for the assessment of LV function, LV ejection fraction may substantially overestimate myocardial performance [4, 5].

However, the risk of functional LV maladaptation, the reaction of right ventricular (RV) function, and the resulting clinical implications after mitral valve surgery for isolated mitral regurgitation are poorly defined [6]. On the other side, evaluation of RV function, particularly after cardiac surgery, is challenging due to the complexity of RV geometry, the high RV sensitivity to hemodynamical changes and ventricular interdependence [7].

Speckle-tracking based myocardial deformation analysis has meanwhile become an established method to evaluate myocardial function. Speckle-tracking based assessment of longitudinal strain is independent of the insonation angle, and can be used retrospectively on digitally archived standard grey-scale images [8].

Hence, we aimed to evaluate LV and RV contractile function using longitudinal strain by speckle-tracking analysis together with the clinical status of patients with isolated PMR before and 3 months after mitral valve surgery.

## Methods

Assessment of exercise tolerance by the New York Heart Association (NYHA) classification alongside, the 6-min walking test and echocardiographic examinations were prospectively performed before and 3 months after surgery in all consecutive patients with severe PMR who underwent isolated mitral valve surgery between July 2015 and May 2017. The decision for surgical treatment was made after heart team discussion for each case individually. The study was approved by local Ethics Committee of Ruhr University of Bochum and carried out in accordance with the Declaration of Helsinki. All data were included in a database, which is registered at www.clinicaltrials.gov (NCT02296710).

### Standard echocardiography

All study participants underwent standard transthoracic echocardiography (EPIQ seven, Philips Electronics, Netherlands). The echo studies were performed by highly qualified medical staff and analysed by the same echocardiographer with long-time experience. The analyses and grading of the mitral regurgitation were performed according to the recommendations of the American and European Societies of Echocardiography [9, 10].

In cases with irregular cardiac rhythm (e.g. atrial fibrillation, frequent atrial or ventricular ectopy) at least five loops were recorded and the average values has been provided.

LV ejection fraction was assessed using the Simpson´s method. LV stroke volume was calculated by subtraction of the LV end-systolic volume from the end-diastolic volume. The Nyquist-limit was placed around 50–60 cm/s in color Doppler settings.

To characterize RV function, tricuspid annular plane systolic excursion (TAPSE) and RV fractional area change (RV-FAC) were measured alongside with RV free wall longitudinal strain analysis.

### Strain analyses

**Fig. 1:**
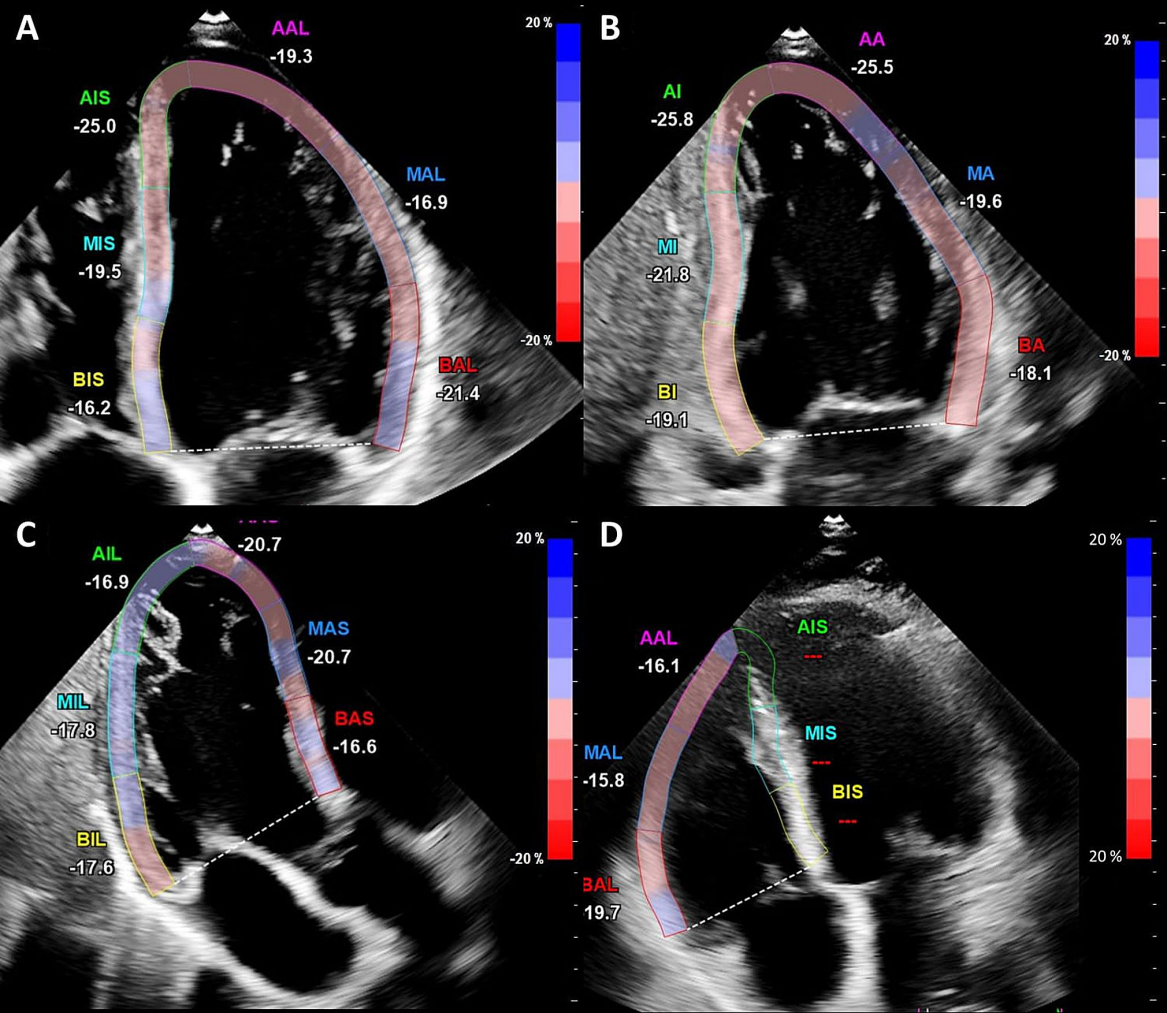
Strain analysis of the left and right ventricle Apical four **a** two **b** three chamber view **c** and a right ventricular focus view **d** were used for strain analysis. An end-diastolic frame was selected for the left ventricle (with the interventricular septum) and for the right ventricular free wall (without the interventricular septum), and the endocardial contour was manually tracked. The other frames were automatically tracked and corrected, if necessary. After verification the longitudinal strain was automatically calculated on average and regionally

LV global longitudinal strain (GLS) was assessed as previously described using the speckle-tracking algorithm provided within the QLAB system (QLAB Version 10.2) [11]. Through three apical views (four-chamber view, three-chamber view, two-chamber view) the end-diastolic frame was selected and the endocardial contour was tracked manually (Fig. 1a-c).

RV free wall longitudinal strain assessment was performed using a RV focused view with optimized RV endocardial borders according to the recommendations of the European and American Societies of Echocardiography (Fig. 1d) [12]. The other frames of the cineloop were tracked automatically and adjusted manually, if needed. Additionally, strain dispersion was documented for each LV segment. Mechanical strain dispersion was calculated as the difference between the highest and the lowest value from time to peak strain assessed through the three apical planes [13].

### Statistical analysis

Statistical analysis was performed using the SPSS-Software (Version 21, IBM Corporation, Armonk, NY, USA). Continuous variables are reported as mean ± standard deviation. Categorical variables are presented as frequencies and percentages. Baseline data were validated for normal distribution using the Kolmogorov–Smirnov method. Student’s T-test for unpaired and paired parametric samples or their analogues for nonparametric samples (Mann–Whitney and Wilcoxon signed rank) or the chi-square test were performed for group comparisons. A p-value < 0.05 was considered significant for all comparisons.

## Results

A total of 156 consecutive patients with primary mitral regurgitation were admitted and evaluated for mitral valve repair between July 2015 and May 2017. Five of them also required myocardial revascularization and four patients presented with a combined valve disease which had to be addressed. eight patients refused participation in the study and one patient was found to suffer from mitral valve endocarditis. Finally, 138 patients were included in the analyses. The baseline characteristics including parameters for mitral regurgitation severity are shown in Tables 1 and 2. Patients’ mean age was 65.8 ± 12.7 years, and 66 (47.8%) of them were female. Mean EuroScore II was 2.6 ± 2.8%, defining a low to intermediate perioperative risk. Mean LV ejection fraction was 57 ± 12%, and degree of mitral regurgitation was characterized by an effective regurgitant volume of 43 ± 3 mm^2^, a regurgitant volume of 67 ± 7 ml and a mean biplane vena contracta of 7.3 ± 0.5 mm. Out of the entire group 95 patients (68.9%) underwent mitral valve repair and 43 (31.1%) valve replacement.

**Table 1:** Collective-wide baseline characteristics

Baseline characteristics (n = 138)	
Age	65.8 ± 12.73 (68)
Female	47.8% (66)
Coronary artery disease	10.9% (15)
Body mass index [kg/m^2^]	26.4 ± 4.3
EuroScore I [%]	8.6 ± 8.5%
EuroScore II [%]	2.6 ± 2.8%
Peripheral artery disease	4.3% (6)
Stroke	11.6% (16)
Diabetes mellitus	8.7% (12)
Renal insufficiency	10.1% (14)
Chronic obstructive pulmonary disease	7.2% (10)
Left bundle branch block	2.2% (3)
History of myocardial infarction	4.3% (6)
History of percutaneous coronary intervention	5.1% (7)
History of cardiac surgery	11.6% (16)
Atrial fibrillation	34.8% (48)
Pacemaker	3.6% (5)

**Table 2:** Baseline echocardiographic parameters of the whole collective

Baseline echocardiographic parameters	
MV PISA radius adjusted to Nyquist limit 30–40 cm/s [mm]	10 ± 3
MR vena contracta [mm]	7.3 ± 0.5
MR effective regurgitant orifice area [mm^2^]	43 ± 3
MR regurgitant volume [ml]	67 ± 7
LA maximal diameter length [mm]	66 ± 12
LA maximal diameter width [mm]	55 ± 1
LA volume [ml]	137 ± 74
LA volume index [ml/m^2^]	72 ± 30
LV EF Simpson [%]	57 ± 12

*MV* mitral valve, *MR* mitral regurgitation, *LA* left atrium, *LV* left ventricle, *EF* ejection fraction, *PISA* proximal isovelocity surface area

Details of echocardiographic parameters before and after surgery are presented in Table 3 (left ventricle) and Table 4 (right ventricle). Three months after surgery, 121 patients (87.7%) had no residual MR and in 17 patients (12.3%) only trivial MR was detectable.

**Table 3:** Morphological and functional changes of the left ventricle

Echocardiographic parameters	Before surgery	3 months after surgery	p-value
LA maximal diameter length [mm]	66 ± 11	55 ± 10	< 0.001
LA maximal diameter width [mm]	55 ± 9	50 ± 8	< 0.001
LA volume [ml]	137 ± 74	97 ± 51	< 0.001
LA volume index [ml/m^2^]	72 ± 30	47 ± 25	< 0.001
LVEDD [mm]	58 ± 7	55 ± 8	< 0.001
LVESD [mm]	4.0 ± 7	4 ± 9	0.5
Septum thickness [mm]	9 ± 2	8 ± 4	0.4
Post wall thickness [mm]	9 ± 2	9 ± 1	0.6
FS [%]	31 ± 9	28 ± 11	0.003
LV end-diastolic volume [ml]	157 ± 57	138 ± 51	< 0.001
LV end-systolic volume [ml]	67 ± 31	70 ± 35	0.2
LV EF Simpson [%]	57 ± 12	50 ± 11	< 0.001
Stroke volume [ml]	93 ± 39	68 ± 25	< 0.001
GLS [%]	−19.2 ± 4.1	−15.7 ± 3.8	< 0.001
Mechanical strain dispersion [msec]	88 ± 12	117 ± 115	0.004

*LA* left atrial, *LV* left ventricular, *LVEDD* left ventricular end-diastolic diameter, *LVESD* left ventricular end-systolic diameter, *FS* fractional shortening, *GLS* global longitudinal strain, *EF* ejection fraction

**Table 4:** Morphologic and functional changes of the right ventricle

Echocardiographic parameters	Before surgery	3 months after surgery	p-value
RA maximal diameter length [mm]	57 ± 11	54 ± 10	0.005
RA maximal diameter width [mm]	45 ± 10	44 ± 8	0.9
RA volume [ml]	78 ± 54	74 ± 45	0.8
RV maximal diameter [mm]	43 ± 9	43 ± 8	0.9
TAPSE [mm]	22 ± 5	18 ± 4	< 0.001
RV end-diastolic area [mm2]	19 ± 6	19 ± 7	0.4
RV end-systolic area [mm2]	11 ± 5	11 ± 4	0.2
FAC [%]	42 ± 12	42 ± 11	0.7
RV basal segment strain [%]	−15.6 ± 5.5	−14.9 ± 4.5	0.3
RV middle segment strain [%]	−18.3 ± 7.0	−16.2 ± 4.7	0.004
RV apical segment strain [%]	−17.1 ± 6.8	−15.9 ± 5.0	0.4
RV mean strain [%]	−16.9 ± 5.6	−15.7 ± 4.1	0.05
Tricuspid regurgitation degree [I-III]	33.6% 0 46.0% I 15.1% II 5.3% III	44.3% 0 50.4% I 4.4% II 0.9% III	< 0.001
TV PISA radius adjusted to Nyquist limit 30–40 cm/s [mm]	3.6 ± 5.0	2.8 ± 4.0	0.04
TR vena contracta [mm]	1.9 ± 0.3	1.1 ± 0.3	0.03

*FAC* fractional area change, *RA* right atrial, *RV* right ventricular, *TAPSE* tricuspid annular plane systolic excursion, *TR* tricuspid regurgitation, *TV* tricuspid valve, *PISA* proximal isovelocity surface area

LV end-diastolic volume markedly decreased from 157 ± 57 ml to 138 ± 51 ml (p < 0.001) following valve surgery, while the other morphological parameters such as end-systolic diameter, septal thickness and posterior wall thickness did not change.

Compared to baseline examinations, LV ejection fraction decreased from 57 ± 12% to 50 ± 11% (p < 0.001) while LV GLS deteriorated from −19.2 ± 4.1% to −15.7 ± 3.8% (p < 0.001). Additionally, strain dispersion increased from 88 ± 12 ms to 117 ± 115 ms (p = 0.004).

**Fig. 2:**
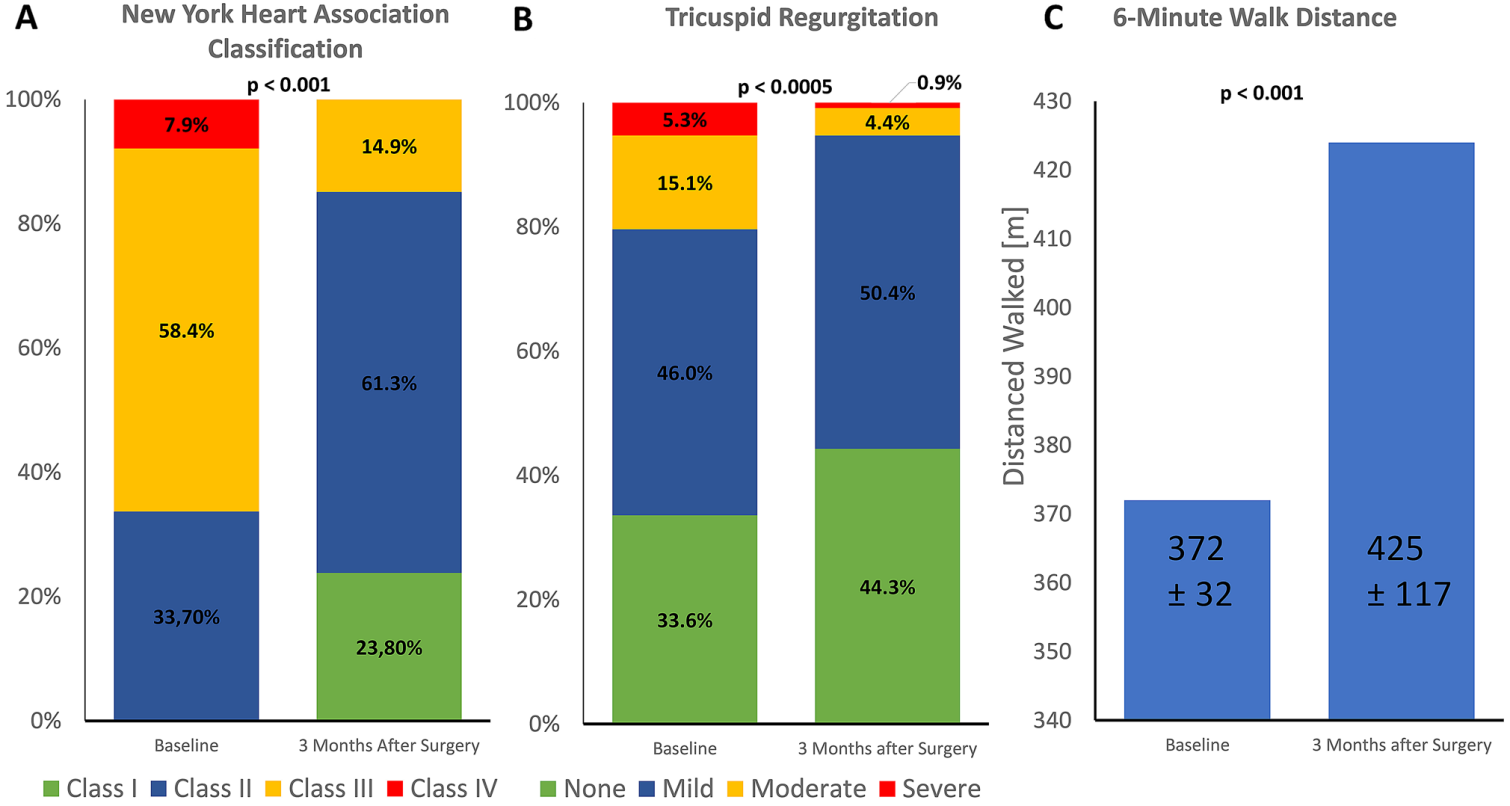
Clinical Improvement and Reduction of Tricuspid Regurgitation after Mitral Valve Surgery Patients with symptomatic primary mitral regurgitation presented a noticeably clinical improvement accompanying the reduction of tricuspid regurgitation as a sign for economized right ventricular function. The New York Heart Association class improved significantly. At baseline 66.3% were in NYHA class III or IV. 3 months after surgery 85.2% were in NYHA class I or II (p < 0.001) **a** The rate of moderate to severe tricuspid regurgitation decreases from 20.4% to 5.3% **b** The walking distance in the 6-min walking test increased from 372 ± 32 m to 425 ± 117 m (p < 0.001) (**c**)

With respect to RV function, tricuspid annular plane systolic excursion (TAPSE) was reduced from 22 ± 5 mm to 18 ± 4 mm (p < 0.001). RV free wall mean strain also showed a slight although borderline significant deterioration (from −16.9 ± 5.6% to −15.7 ± 4.1%; p = 0.05). However, tricuspid regurgitation improved after mitral valve surgery (Fig. 2b). While 20.4% of the patients had a moderate to severe TR before mitral valve surgery, this figure decreased to 5.3% 3 months after surgery with consecutively decreasing RA diameter (Table 4).

Regarding exercise tolerance, NYHA classification (at baseline 66.3% were in NYHA class III or IV, 3 months after surgery 85.2% were in NYHA class I or II; p < 0.001) and walking distance in the 6-min walking test (372 ± 32 m to 425 ± 117 m; p < 0.001) improved significantly (Fig. 2a, c).

## Discussion

Due to the poorly defined risk for ventricular dysfunction after mitral surgery and its clinical impact, we evaluated the adaptation of the left and right ventricle after surgical mitral valve treatment in patients with severe mitral regurgitation and the clinical status before and 3 months after surgery.

### Left ventricular dysfunction after mitral valve surgery

Mean LV GLS in our patients was −19.2% at baseline and showed a deterioration after mitral valve surgery as an indicator for LV dysfunction. This is in accordance with the retrospective observation of Witkowski et al. who described a GLS worse than −19.9% as an independent predictor for LV dysfunction in severe primary mitral regurgitation [14].

Hiemsatra et al. described LV GLS as independently associated with all-cause mortality and cardiovascular events in a cohort of 593 patient who underwent mitral valve surgery with a median follow-up of 6.4 years, (Hazard ratio 1.13; 95% confidence interval: 1.06 to 1.21 p < 0.001). In this study, LV-EF and LV GLS showed a similar deterioration of the contractile function (3). In a retrospectively analysed cohort of 506 patients with a wide range of cardiac comorbidities and a median follow-up of 3.5 years, Kim et al. postulated GLS to better predict cardiac events and all-cause mortality than standard echocardiographic parameters (Multivariate Cox models HR 1.229 95% CI: 1.135 to 1.331; p < 0.001). The authors concluded this measure to be helpful to estimate the optimal timing for mitral valve surgery [15]. Interestingly, mechanical strain dispersion also increases after mitral valve surgery (Table 4). Prolonged mechanical strain dispersion is a sign for heterogeneity of systolic myocardial contraction due to the development of fibrosis formation and is associated with cardiac arrhythmias [16]. Therefore, strain dispersion could provide important information about cardiac remodeling during patient evaluation for mitral valve surgery [17].

However, despite functional impairment of the left ventricle, the patients showed pronounced clinical improvement in NYHA class and 6-min walking distance (Fig. 2a, c). Moreover, LA and LV diameter and volumes decreased after mitral valve surgery demonstrating a relevant reverse remodelling. By eliminating the regurgitation fraction of overall stroke volume, LV enlargement receded allowing for normal stress shortening [18, 19]. However, since stroke volume and ejection fraction are required for antegrade flow only, myocardial performance is optimized and economized [20] whereas, according to our results, at least a temporary postoperative medical therapy to support myocardial unloading and reverse remodelling is suggested.

### Right ventricular dysfunction after mitral valve surgery

Mitral regurgitation leads to a volume overload of the LA [18]. The LA is initially able to keep the pressure stable through its enlargement, but over time the pressure in the pulmonary venous system increases which eventually leads to an increased pulmonary artery pressure [7].

In the absence of volume overload after surgery, the pressure in the pulmonary vascular bed and consecutively in the right ventricle decreases. Right ventricular dimensions and functional tricuspid regurgitation are consecutively reduced [7]. However, as on the left side, some measures of RV function decreased. While FAC did not change, RV free wall strain and TAPSE were reduced. This deterioration is probably explained by geometric changes of the RV due to pericardial incision and the loss of pericardial support [21]. Depending on the pericardial incision and the surgical access path, parameters for the longitudinal RV function can show a decrease, despite overall normal global right ventricular function [21].

Another aspect is the reduced mobility of the septum due to the increased LV impairment. In addition, the incompletely understood cardioplegia effect may have played a role [3, 22–24]. The septal wall is involved in the mechanism of “squeezing out” the right ventricle. Together with the apex, the septal wall serves as an abutment to counteract the tension of the bellow-type right ventricle, and thus transports the blood towards the pulmonary arteries. About 24% of the RV function is taken over by the septal wall [7].

Our mid-term follow-up data on exercise tolerance demonstrate a clear clinical improvement, which implies an economization and higher effectiveness of RV myocardial performance. Accordingly, tricuspid regurgitation also improved after surgery probably because of improved hemodynamic and lack of volume overload which is also a sign of recovered clinical status [25, 26].

### Limitations

The study is descriptive and not designed to explain the phenomena it observes and can therefore only generate hypotheses. In addition, further studies should investigate whether and to what extent the deteriorated function parameters persist during longer-term follow-up and whether this has a long-term impact on survival.

## Conclusion

During mid-term follow up after surgery for PMR, a deterioration of LV and RV contractile function measures could be observed. However, the clinical status, LV dimensions, and concomitant tricuspid regurgitation improved significantly which in particular imply more effective RV contractile pattern.

## Data Availability

All presented data are available and will be issued if necessary.
